# Identification and Validation of Carbonic Anhydrase II as the First Target of the Anti-Inflammatory Drug Actarit

**DOI:** 10.3390/biom10111570

**Published:** 2020-11-19

**Authors:** Ghita Ghislat, Taufiq Rahman, Pedro J. Ballester

**Affiliations:** 1 Centre d’Immunologie de Marseille-Luminy, Inserm, U1104, CNRS UMR7280, F-13288 Marseille, France; 2 Department of Pharmacology, University of Cambridge, Cambridge CB2 1PD, UK; mtur2@cam.ac.uk; 3 Centre de Recherche en Cancérologie de Marseille (CRCM), Inserm, U1068, F-13009 Marseille, France; 4 CNRS, UMR7258, F-13009 Marseille, France; 5 Institut Paoli-Calmettes, F-13009 Marseille, France; 6 Aix-Marseille University, UM 105, F-13284 Marseille, France

**Keywords:** actarit, carbonic anhydrase II, malotilate, MolTarPred, target prediction

## Abstract

**Background and purpose:** Identifying the macromolecular targets of drug molecules is a fundamental aspect of drug discovery and pharmacology. Several drugs remain without known targets (orphan) despite large-scale in silico and in vitro target prediction efforts. Ligand-centric chemical-similarity-based methods for in silico target prediction have been found to be particularly powerful, but the question remains of whether they are able to discover targets for target-orphan drugs. **Experimental Approach:** We used one of these in silico methods to carry out a target prediction analysis for two orphan drugs: actarit and malotilate. The top target predicted for each drug was carbonic anhydrase II (CAII). Each drug was therefore quantitatively evaluated for CAII inhibition to validate these two prospective predictions. **Key Results:** Actarit showed in vitro concentration-dependent inhibition of CAII activity with submicromolar potency (IC_50_ = 422 nM) whilst no consistent inhibition was observed for malotilate. Among the other 25 targets predicted for actarit, RORγ (RAR-related orphan receptor-gamma) is promising in that it is strongly related to actarit’s indication, rheumatoid arthritis (RA). **Conclusion and Implications:** This study is a proof-of-concept of the utility of MolTarPred for the fast and cost-effective identification of targets of orphan drugs. Furthermore, the mechanism of action of actarit as an anti-RA agent can now be re-examined from a CAII-inhibitor perspective, given existing relationships between this target and RA. Moreover, the confirmed CAII-actarit association supports investigating the repositioning of actarit on other CAII-linked indications (e.g., hypertension, epilepsy, migraine, anemia and bone, eye and cardiac disorders).

**Bullet Point Summary**

*What Is Already Known:*
  - Computational target prediction methods complement and/or guide experimental approaches to characterise the polypharmacology of drugs.
  - Ligand-centric chemical-similarity-based methods (e.g., MolTarPred, freely available at http://moltarpred.marseille.inserm.fr/) have been found to be particularly powerful.
*What This Study Adds:*
  - Experimental confirmation of MolTarPred prediction of CAII as a target of Actarit with a mid-nanomolar IC_50_.
  - A proof-of-concept of MolTarPred’s utility for target-orphan drugs, providing other plausible target predictions for actarit (e.g., RORγ).
*Clinical Significance:*
  - The CAII-actarit association sheds light into its mechanism of action as a drug for RA (rheumatoid arthritis).
  - Repositioning of actarit can now be investigated for other CAII-linked indications.

## 1. Introduction

Discovering the molecular targets of a molecule is important for its potential use as a drug [1,2]. For example, knowing the targets of a drug lead with phenotypic activities is useful to position the lead on a given indication or to identify its potential toxicities. For drugs already approved for clinical use, discovering a new target permits improving our understanding of the mechanism of action of the drug or even repositioning it into an unexpected indication associated with that target.

Studies including reviews of computational methods for target prediction have been published [3,4]. These methods have shown their utility for tackling these problems, both retrospectively [5,6] and prospectively [7,8,9]. An early example of these methods is the Similarity Ensemble Approach (SEA), which constructs a statistical model for each considered target and uses the ensemble of models to predict which targets interact with the investigated molecule.

SEA has been prospectively applied to de-orphanize drugs without known protein targets [7]. More concretely, these authors analysed a set of 1431 world-wide approved drugs. They found that 1079 of these drugs have known targets in bioactivity databases such as ChEMBL. The remaining 352 drugs were analysed with SEA, which managed to identify targets for 308 of these drugs (mostly the SEA-suggested target could be verified in the literature, but also experimentally confirmed in some cases). Despite these efforts, targets could not be found for a set of 41 drugs listed in their Table S4 [7].

Methods based on other principles may be able to provide useful predictions for these hard-to-predict target-orphan drugs. In particular, target prediction methods based on chemical similarity have been found to be particularly powerful [6]. We developed MolTarPred [4], a chemical-similarity-based method able to predict more than 4500 protein targets, and made it freely available as a webserver [10]. This version of MolTarPred has recently predicted new targets for mebendazole and nocodazole [9]. These predicted targets were confirmed in vitro: mebendazole inhibit MAPK14 with an IC_50_ of 104 nM and nocodazole inhibit ABL1 with an IC_50_ of 78 nM [9]. Although several targets were already known for these two anti-helminthic drugs, the discovery of their potent activity against these cancer targets led to a better explanation of their anti-glioma properties [9]. 

Here we present a proof-of-concept study showing that MolTarPred can also be useful with hard-to-predict target-orphan drugs. In particular, MolTarPred revealed that one of these target-orphan drugs (actarit) has potent activity against human carbonic anhydrase II (CAII). This discovery may be helpful to understand the mechanism of action of actarit in rheumatoid arthritis (RA) and opens the door to repositioning this anti-inflammatory drug to other CAII-linked indications.

## 2. Materials and Methods

### 2.1. MolTarPred (Molecular Target Prediction) 

MolTarPred is a user-friendly web tool for predicting the targets of small organic molecules [10]. In a nutshell, MolTarPred calculates the similarities between the query molecule (i.e., the molecule for which predicted targets are sought after) and many thousands of molecules (the knowledgebase), each knowledgebase molecule with at least one target annotated. Then, each target annotated in the 10 most similar molecules is predicted as a target of the considered query molecule. MolTarPred provides an intuitive estimate of the reliability of each target predicted for a given query molecule [10], which also has the advantage of being validated [4]. The reliability of a predicted target is the number of top hits that have that target annotated, thus this metric ranges from 1 (minimum reliability) to 10 (maximum reliability). This ligand-centric approach provides by construction the maximum coverage of target space for a given dataset [4]. The first version (v1) of this tool exploited a knowledgebase with 184,912 molecules retrieved from release 20 of the ChEMBL database [11] and thus were annotated with 3046 single-protein targets (a molecule is annotated with a target if the K_i_, K_d_, IC_50_ or EC_50_ of the pair is lower than 10 μM). The second version (v2) of MolTarPred is the current version online (http://moltarpred.marseille.inserm.fr/) and that was thoroughly described in a recent publication [10]. MolTarPred v2 exploits a much larger knowledgebase than v1 comprising 607,659 small-molecule ligands annotated with at least one of 4553 protein targets [10]. A comprehensive description of how to use MolTarPred and interpret its results has already been published [10], with two previous papers developing and validation its working principles [4,5].

### 2.2. CAII Activity Assay

CAII activity was measured using Wilbur and Anderson’s electrometric method [12]. Briefly, the time required for a saturated carbon dioxide solution to lower the pH of a 20 mM Trizma buffer from 8.3 to 6.3 at 0 °C was determined without (T_0_) or with 0.02 mg/mL of CAII +/− inhibitors (T) to calculate the Wilbur-Anderson unit (2 × (T_0_ − T))/T of each sample. The activity of CAII was assessed as Wilbur-Anderson units/mg of CA II in each reaction. 

### 2.3. Materials

The following reagents were purchased from Sigma-Aldrich (Saint-Quentin-Fallavier, France): recombined human carbonic anhydrase II (C6165), Trizma buffer (T1501) and pH 4, 7 and 10 standard buffers (B5020, B4770 and B4895). Actarit (95% purity) and malotilate (95% purity) were purchased from Molport (MolPort-002-468-862 and MolPort-006-131-867, respectively (Molprot, Riga, Latvia), and were supplied by Enamine Ltd. (EN300-13165, Enamine Ltd., Kiev, Ukraine) and AK Scientific, Inc. (C865, AK Scientific, Inc., Union City, CA, USA), respectively.

## 3. Results

The analysis of the 41 target-orphan drugs was carried out using MolTarPred v1, before v2 was available. More concretely, for this proof-of-concept, we non-exhaustively searched for a target predicted on at least 2 of the 41 drugs. This strategy is intended to optimise our resources, as in vitro evaluation of target predictions can be technically very demanding due to the need for a distinct experimental approach per target. For example, the evaluation of five molecules predicted to have the same target is much easier than that of one molecule predicted to interact with five targets, despite each case requiring the evaluation of five ligand-target activity values. This is because the former case requires setting up only 1 assay while the latter needs having 5 different assays in place. On the other hand, we were interested in target predictions with the highest estimated reliability for the drug to maximize the likelihood of the predicted target being a true target. Lastly, we requested predicted targets linked to disease indications via drugs approved for patient use. In this way, any new molecular target would not only provide clues about the mechanism of action of the target-orphan drug, but also unveil new repositioning opportunities for the drug. 

**Figure 1:**
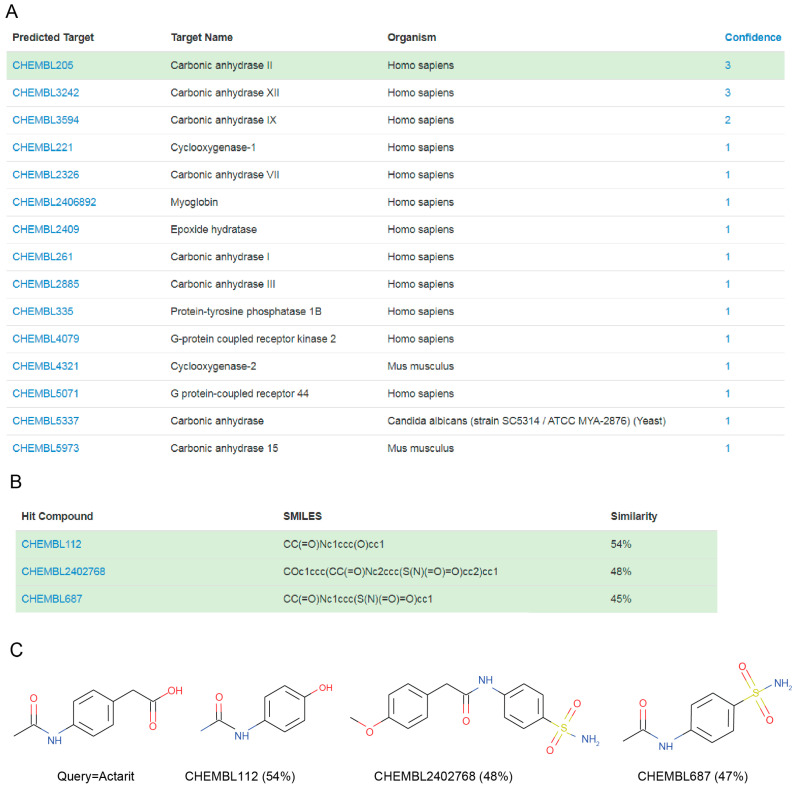
(**A**) MolTarPred v1′s predicted targets for actarit. There are 15 predicted targets, one per row, sorted by decreasing reliability (confidence) score. Carbonic anhydrase II (CAII), highlighted in green, appears as a target of actarit with a reliability score of 3. (**B**) SMILES and similarity scores of the three top hits annotated with the inspected predicted target (CAII). (**C**) Chemical structures of the query molecule (actarit; left) and its three CAII-annotated top hits listed in (**B**) on the right.

**Figure 2:**
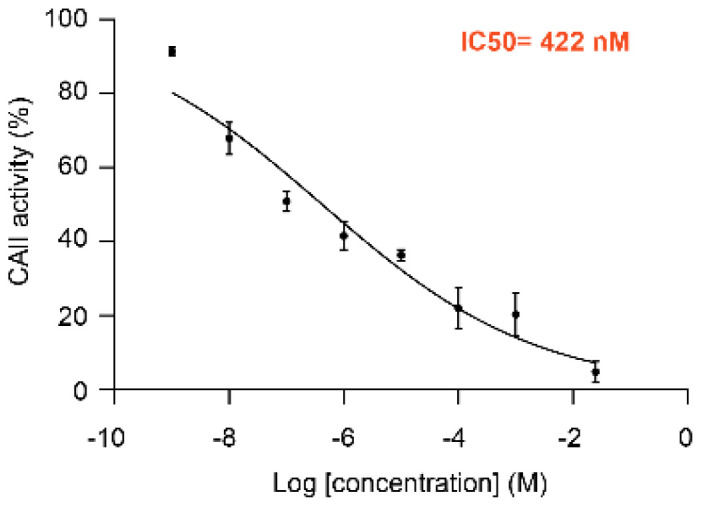
Dose-response curve and IC_50_ value of CAII inhibition by actarit (IC_50_ of 422 nM). Error bars represent the standard error of the mean of triplicate average values. Data were fit by non-linear regression (R^2^ = 0.94138) and IC_50_ values were determined using the equations Y = 100/(1 + 10^((LogIC_50_ − X)*HillSlope)) where Y is the percentage of CAII activity relative to untreated samples, using GraphPad Prism 7.00 software.

MolTarPred v1 revealed human CAII as a target with the sought characteristics, which was predicted for two of the target-orphan drugs: actarit and malotilate. Actarit is a disease-modifying anti-rheumatic drug, or DMARD [13,14], that can be orally administered [15]. Figure 1 shows the targets predicted for actarit by MolTarPred v1, with CAII being predicted with the highest reliability (Figure 1A). CAII was predicted with reliability 3, as 3 of the top 10 most similar molecules to actarit were annotated with this target (Figure 1B,C). A target predicted with reliability 3 is estimated to be a true target of the drug 41.7% of the time [4]. To experimentally validate whether or not CAII could be a target for actarit, we quantitatively evaluated actarit against the activity of recombinant human CAII in vitro following the established method [12]. As observed in Figure 2, actarit showed concentration-dependent inhibition of CAII activity with submicromolar potency (IC_50_ = 422 nM).

**Figure 3:**
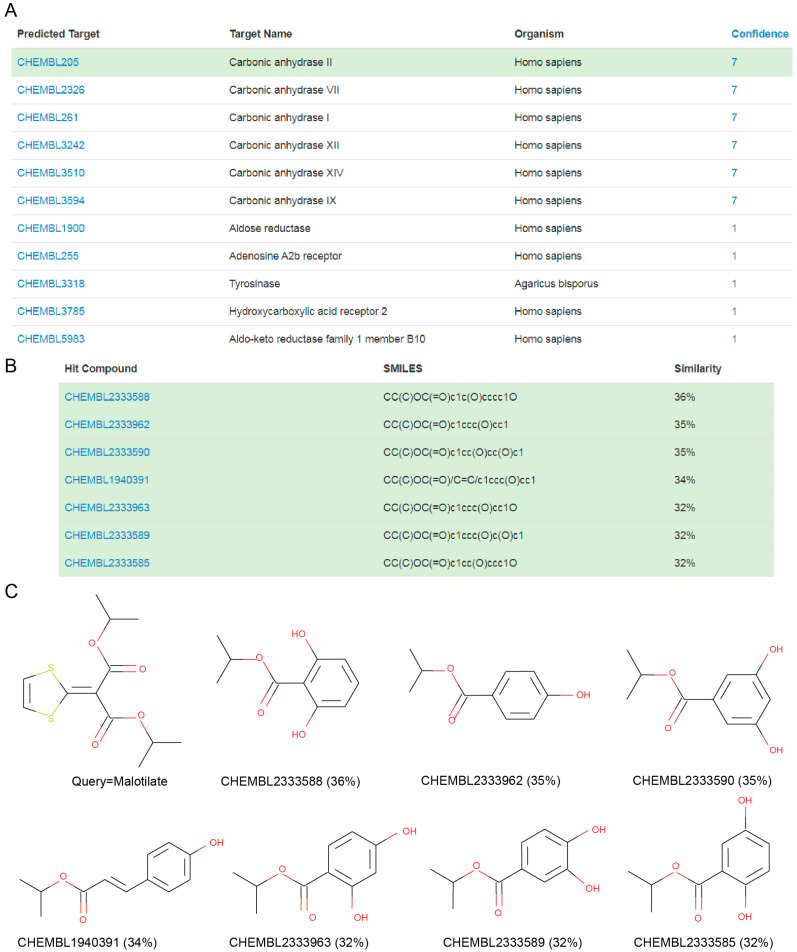
(**A**) MolTarPred v1′s predicted targets for malotilate. There are 11 predicted targets, one per row, sorted by decreasing reliability (confidence) score. CAII, highlighted in green, appears as a target of malotilate with a reliability score of 7. (**B**) SMILES and similarity scores of the seven top hits annotated with the inspected predicted target (CAII). (**C**) Chemical structures of the query molecule (malotilate; top left) and its seven CAII-annotated top hits listed in (**B**) to its right and bottom row.

**Figure 4:**
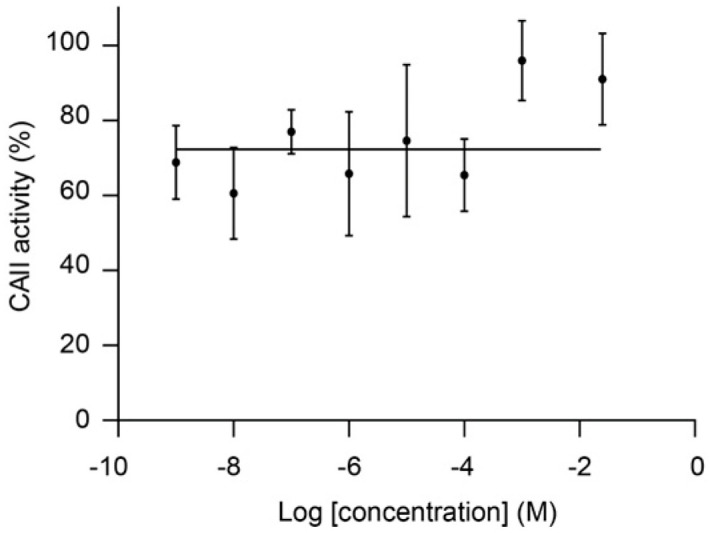
CAII inhibition by malotilate lacks a dose-response curve and thus data could not be well fitted by non-linear regression to calculate an IC_50_. Error bars represent the standard error of the mean of triplicate average values.

CAII was also the target predicted with highest reliability for malotilate, an orally-administrated drug for the treatment of hepatic diseases [16]. Figure 3 shows that the targets predicted for malotilate by MolTarPred v1. CAII were predicted with reliability 7 (Figure 3A), as 7 of the top 10 most similar molecules to malotilate were annotated with this target (Figure 3B,C). To validate the observed CAII-malotilate prediction, we tested malotilate against human CAII activity using an aforementioned quantitative approach, but observed no consistent inhibition in vitro (Figure 4). The lack of a dose-response curve meant that its IC_50_ could not be calculated. 

## 4. Discussion and Conclusions

Despite substantial research on actarit, e.g., exploring its clinical phenotype [15,17], improving its administration [18,19] or enhancing its stability within the organism [20,21], no molecular target of this drug has previously been found. MolTarPred v1 was hence able to discover the first target for actarit where SEA could not [7]. Running the current version of SEA (http://sea.bkslab.org/) on actarit returns CAII as the 74th predicted target and not being ranked sufficiently high seems the reason why this target-drug association was missed. ChEMBL recently supplements their compiled measurements with in silico target predictions [22], which also missed this target of actarit (https://www.ebi.ac.uk/chembl/compound_report_card/CHEMBL1885632). This shows that MolTarPred can complement these methods for in silico target prediction analysis. 

Relationships between some carbonic anhydrase (CA) proteins and RA have been reported. Pan-CA inhibitors, including CAII, were previously shown to be efficient for the treatment of RA [23,24]. CAIII and CAIV autoantibodies, which inhibit these CA activities, were identified as diagnostic markers of RA [25,26]. In line with these observations, CAII autoantibodies were as well found in high amounts in RA patients where a correlation with oxidative stress was observed [27]. Thus, CAII may be the primary target of actarit for the treatment of RA. CAII is a zinc-dependent metalloenzyme. The carboxylate group of actarit may serve as a zinc-chelating agent blocking the catalytinc activity of CAII. We stipulate that the discovery of CAII as a molecular target of actarit will help to gain a better understanding of the mechanism of action of this drug. On the other hand, the molecular function of CAII can have broader applications. This enzyme target catalyses the reversible hydration of carbon dioxide, which dissociates into protons, and bicarbonate ions in a widespread fashion among tissues and cell compartment across many organisms [28]. A balance between carbon dioxide and protons/bicarbonate is essential for a plethora of physiological processes and excess of insoluble protons/bicarbonate may destabilize physiological pH. Thus, dysfunctions in the enzymatic activity or expression level of CAII have been associated with a range of diseases, from anemia [29] and bone, eye and cardiac disorders [30] to various cancers [31]. Some examples of FDA-approved CAII inhibitors are brinzolamide to treat elevated intraocular pressure, chlorothiazide indicated for hypertension and topiramate to treat epilepsy and migraines. Therefore, our study could also contribute to expand the indications of actarit. The of actarit, as a human-safe CAII inhibitor can now be investigated in the same indications associated with CAII.

MolTarPred also has the advantage of offering interpretability for its predictions. For example, the most similar molecule to actarit was CHEMBL112, also known as paracetamol (Figure 1B), with both chemical structures only differing in a terminal functional group (Figure 1C). Paracetamol has moderate CAII activity with a K_i_ of 6.2 μM [32]. This means that MolTarPred v1 was able to identify a new CAII inhibitor with about 15-fold higher potency (IC_50_ of 422 nM) than its closest top hit and that the functional group substitution (carboxymethyl in actarit, hydroxyl in paracetamol) is the cause of such potency enhancement. Furthermore, given the high similarity between actarit and paracetamol, it is likely that actarit also has similar activity to at least some of the other targets of paracetamol. This merits further investigation and might lead to further repositioning opportunities.

Like actarit, malotilate was classified as a hard-to-predict target-orphan drug [7] and the ChEMBL database does not report any known target either (https://www.ebi.ac.uk/chembl/compound_report_card/CHEMBL1697754). However, there is a 30-year-old study reporting an IC_50_ of 4.7 μM with 5-lipoxygenase [33]. This target was not only missed by SEA, but also MolTarPred and the ChEMBL method. A possible reason is that the activity of this association is close to the threshold of activity of these methods (10 μM). 

By comparing Figure 1 and Figure 3, one can see that CAII was predicted for malotilate with higher reliability than for actarit, yet no CAII inhibition was induced by malotilate. There are several factors that could explain this opposite outcome. First, higher reliability only confers a higher likelihood of being a true target [4], so while less likely the result is entirely possible. Second, target inference was carried out from less similar top hits in malotilate (similarity scores between 32% and 36%) than in Actarit (similarity scores ranging from 45% to 54%). By the molecular similarity principle [34], the more similar the molecules are, the higher the likelihood that they hit the same targets. Third, malotilate presents ester groups susceptible to undergo hydrolysis by esterases present in the cytosol, which hints the possibility of this drug being actually a prodrug, in which case any target prediction method would need to consider instead its pharmacologically active form after metabolism. Lastly, there are also more CAII binding sites apart from its active site [35] and hence malotilate could still be binding to CAII without affecting its enzymatic activity. In other words, malotilate-CAII could not be a false positive.

**Figure 5:**
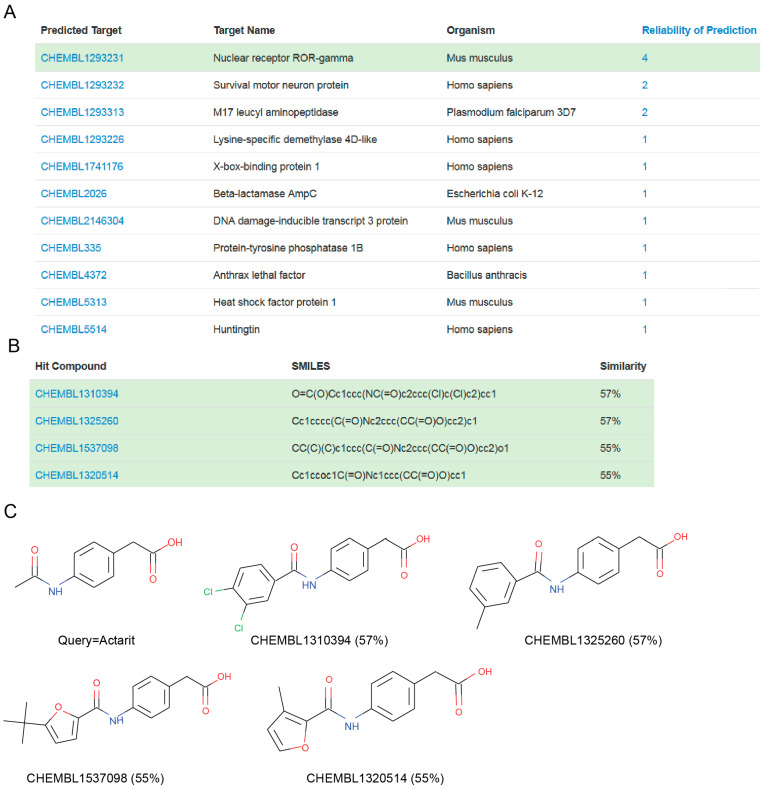
(**A**) MolTarPred v2′s predicted targets for actarit. There are 11 predicted targets, one per row, sorted by decreasing reliability score. ROR-γ, highlighted in green, appears as a target of actarit with a reliability score of 4. (**B**) SMILES and similarity scores of the four top hits annotated with the inspected predicted target (ROR-γ). (**C**) Chemical structures of the query molecule (actarit; top left) and its top four ROR-γ-annotated hits listed in (B) to its right and bottom row.

A way to obtain higher similarities between the chemical structures of a query molecule and its most similar molecules is employing a larger knowledgebase. This will increase the average accuracy of the predictions, which may in turn result in other predicted targets worth considering. For example, if we use instead MolTarPred v2, which has a 3.3-times larger knowledgebase than that of v1, the target predicted with the highest reliability is now murine nuclear receptor ROR (RAR-related orphan receptor)-gamma (RORC or RORγ, for short). Figure 5 shows the four top hits supporting this prediction (reliability 4), with higher similarity scores to actarit (from 55% to 57%) than the top hits from MolTarPred v1 (from 45% to 54%). None of the molecules that were supporting the CAII prediction in MolTarPred v1 are among the top 10 hits in MolTarPred v2 (they are now lower in the ranking) and none of these top 10 hits in MolTarPred v2 are known to bind CAII. As a result, CAII is not predicted as a target of actarit by MolTarPred v2. This seemingly shocking outcome is not unexpected. A high rate of false negatives, i.e., low recovery rates, is a well-known limitation of in silico target prediction, which instead excels at quickly providing target predictions with generally few false positives [5,6], as it has been the case here. This high-false-negative limitation is largely due to sparse nature of ligand-target bioactivity data (even in the most comprehensive databases, only 0.04% of all the possible ligand-target pairs in the knowledgebase have at least one bioactivity value associated [4]). Given the relatively high similarity of the MolTarPred v2′s top hits to actarit (Figure 5), each containing a CAII-privileged carboxylate group, it is very likely that at least some of them will inhibit CAII activity as well, once they are tested in vitro. Updating MolTarPred with any of these top hits as a CAII inhibitor would lead to CAII being predicted again.

RORγ is largely conserved between mice and humans (88.9% amino acid sequence identity). The latter, along with the prediction of murine RORγ, strongly suggests that human RORγ could be another target of actarit, a target that is also related to RA. Indeed, RORγ (the T cell specific isoform is RORγt) is a key transcription factor driving Th17 cell differentiation leading to subsequent production of IL-17A and few other pro-inflammatory cytokines as well as triggering NLRP3 inflammasome activity [36,37]. In addition, hyperactive Th17 cells have been implicated in the pathology of several autoimmune diseases, including multiple sclerosis, RA, psoriasis and Crohn’s disease. [38,39]. Accordingly, genetic ablation of RORγ or its pharmacological inhibition has been shown to be protective in various animal models of some of these autoimmune diseases [37,40]. Thus, actarit is potentially repurposable for these autoimmune diseases.

Overall, this study has predicted 26 targets for actarit (15 from Figure 1 plus 11 from Figure 5). We have evaluated the most reliable prediction in Figure 1, which was found to be correct (CAII-actarit interact in vitro with an IC_50_ of 422 nM). This constitutes a proof-of-concept that MolTarPred can discover targets even for drugs without previously known targets. Furthermore, the mechanism of action of actarit as an anti-RA agent can now be re-examined from a CAII-inhibitor perspective given the explained relationships between CA and RA. Moreover, the confirmed CAII-actarit association supports investigating the repositioning of actarit in CAII-linked indications (e.g., hypertension, epilepsy, migraine, anemia, bone, eye and cardiac disorders, among others).

The provided target prediction analysis permits others to evaluate any of these 25 potential targets of actarit. This is worthwhile because a small-molecule drug hits over 11 targets with an IC_50_ better than 10 µM on average [4] and thus Actarit is likely to hit several other targets. Particularly promising is the predicted RORγ-actarit association, given that RORγ is strongly related to auto-immune diseases, including RA [36].

## Abbreviations

CAII	Carbonic anhydrase II
FDA	Food and Drug Administration
MolTarPred	Molecular Target Prediction
RA	Rheumatoid Arthritis
RORγ	RAR-related orphan receptor gamma
SEA	Similarity Ensemble Approach
